# Retinal Thickness Measurement Obtained with Spectral Domain Optical Coherence Tomography Assisted Optical Biopsy Accurately Correlates with *Ex Vivo* Histology

**DOI:** 10.1371/journal.pone.0111203

**Published:** 2014-10-31

**Authors:** Lee R. Ferguson, Sandeep Grover, James M. Dominguez II, Sankarathi Balaiya, Kakarla V. Chalam

**Affiliations:** 1 Department of Ophthalmology, University of Florida College of Medicine, Jacksonville, Florida, United States of America; 2 Department of Pharmacology and Therapeutics, University of Florida College of Medicine, Gainesville, Florida, United States of America; Justus-Liebig-University Giessen, Germany

## Abstract

**Background:**

This study determines ‘correlation constants’ between the gold standard histological measurement of retinal thickness and the newer spectral-domain optical coherence tomography (SD-OCT) technology in adult C57BL/6 mice.

**Methods:**

Forty-eight eyes from adult mice underwent SD-OCT imaging and then were histologically prepared for frozen sectioning with H&E staining. Retinal thickness was measured via 10x light microscopy. SD-OCT images and histological sections were standardized to three anatomical sites relative to the optic nerve head (ONH) location. The ratios between SD-OCT to histological thickness for total retinal thickness (TRT) and six sublayers were defined as ‘correlation constants’.

**Results:**

Mean (± SE) TRT for SD-OCT and histological sections was 210.95 µm (±1.09) and 219.58 µm (±2.67), respectively. The mean ‘correlation constant’ for TRT between the SD-OCT and histological sections was 0.96. The retinal thickness for all sublayers measured by SD-OCT vs. histology were also similar, the ‘correlation constant’ values ranged from 0.70 to 1.17. All SD-OCT and histological measurements demonstrated highly significant (p<0.01) strong positive correlations.

**Conclusion:**

This study establishes conversion factors for the translation of *ex vivo* data into *in vivo* information; thus enhancing the applicability of SD-OCT in translational research.

## Introduction

Spectral-domain optical coherence tomography (SD-OCT) is an important imaging modality, in clinical ophthalmology and animal research, for characterizing morphology and understanding pathophysiological changes in the retina. The rapid, high resolution, non-invasive cross sectional images produced by SD-OCT is an important tool in diagnosing posterior segment pathology and monitoring it longitudinally [1], [2]. Although SD-OCT provides non-invasive histological–grade sections of the rodent posterior segment, image acquisition is technologically cumbersome as human devices are retrofitted for animal use.

Histological evaluation of retinal tissue in animal models has traditionally been the primary method for investigating the microstructure of the retina. Retinal tissue histology has contributed to our understanding of retinal cellular mechanisms and disease pathophysiology. With advancements in OCT technology, the *ex vivo* histological methodology is being replaced with the more innovative SD-OCT *in vivo* ‘optical biopsy’ [3]. SD-OCT avoids the inherent limitations of the time-consuming, destructive, and cumbersome histological procedures. Moreover, high resolution detailed images comparable to mid- to high-range microscope objective lens is attained with newer generation OCT technology [4]. Additionally, volumetric data can be generated which would provide more insight into structural changes at the microscopic level than visualizing two-dimensional anatomical sections [5].

In this study, we measured retinal thickness non-invasively with SD-OCT optical biopsy and compared it to measurements obtained with histological sections from the same eye and similar anatomical location after sacrificing the animals. We established a correlation constant between SD-OCT retinal thickness and histological retinal thickness. Such a constant can then be applied to estimate the retinal thickness as well as volume in a variety of diseases longitudinally.

## Methods

### Animals

Healthy adult C57BL/6 mice (Jackson Laboratory, Bar Harbor, ME), between the ages of 3 to 5 months, were used for the study. All mice were maintained under a 12-hour light/dark schedule with unrestricted access to food and water at the University of Florida animal care services (ACS) facility. All procedures performed on the mice were implemented in an ACS-authorized location with study approval acquired from the University of Florida institutional animal care and use committee. In addition, the guidelines set by the Association for Research in Vision and Ophthalmology Statement for the Use of Animals in Ophthalmic and Vision Research were followed during experimentation on animal subjects.

### SD-OCT Imaging and Analysis

SD-OCT imaging was performed on the right eye of all animals. The Bioptigen spectral-domain ophthalmic imaging system (Bioptigen, Inc., Durham, NC) was used to capture SD-OCT images. The mice were secured and scanned with the use of the animal imaging mount and rodent alignment stage apparatus [6]. This device allowed for multi-axial manipulation of the animal in order to properly align the mouse eye with the SD-OCT probe. As a result, rapid and reproducible acquisition of the retinal scans, centered at the optic nerve head (ONH), was possible. Mice were anesthetized with an intraperitoneal mixture of ketamine (Ketaject; 80 mg/kg; Webster Veterinary, Devens, MA) and xylazine (Ana Sed; 10 mg/kg; Webster Veterinary; Devens, MA). Topical tropicamide (1%; Akorn Inc.; Lake Forest, IL) was used to dilate the pupils. Corneal desiccation was prevented by applying topical Systane Ultra lubricant eye drop (Alcon, Fort Worth, TX) every minute during the procedure.

**Figure 1:**
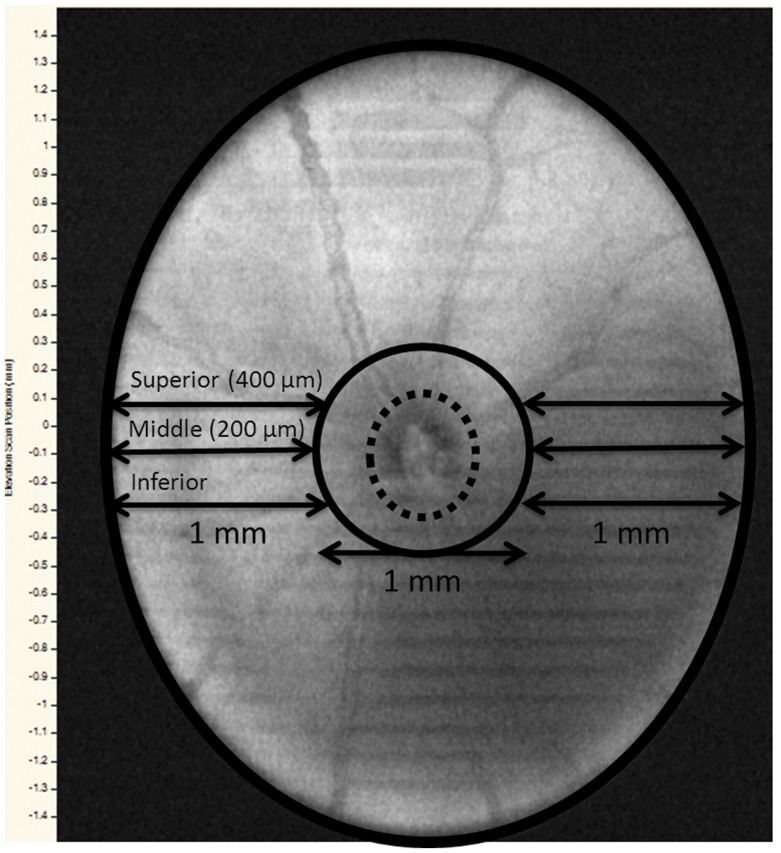
C57BL/6 mouse retina showing the reference points for SD-OCT and histological measurements. Dotted circle – optic nerve head (ONH); black solid circle – 1 mm diameter central area surrounding the ONH; inferior, middle and superior reference points, each 200 µm apart, both to the left and the right of the ONH.

SD-OCT images were obtained with the InVivoVue Clinic software (Bioptigen, Inc., Durham, NC). Briefly, a 3×3 mm perimeter scanning protocol was used to obtain an imaging sequence comprising of 100 B-scans, with each B-scan consisting of 1000 A-scans, through a 50-degree field of view from the mouse lens. Once the ONH was centered within the InVivoVue Clinic imaging application, three scanning sequences were acquired. Measurements were restricted to retinal regions outside a radius of 500 µm from the center of the ONH. Retinal layer measurements were performed via the automated segmentation software provided by the instrument manufacturer (Bioptigen, Inc., Durham, NC). All layers were measured except for the retinal pigment epithelial layer because of the extensive artifactual changes associated with histological preparation and the limited depth penetrance associated with the SD-OCT 4.5 µm axial resolution. Total retinal thickness (TRT) represented the summation of all retinal layers spanning from the retinal nerve fiber layer (RNFL) to the outer segment of photoreceptors/inner segment of photoreceptors/external limiting membrane (OS/IS/ELM) region. Retinal sublayer measurements consisted of the OS/IS/ELM, outer nuclear layer (ONL), outer plexiform layer (OPL), inner nuclear layer (INL), inner plexiform layer/ganglion cell (IPL/GC), and the RNFL. In order to standardize retinal thickness measurements from SD-OCT scans and histology sections, three arbitrary points were selected. The “inferior point” was the reference point that passed through the inferior margin of the ONH. The “middle point” was designated as the B-scan 200 µm above the “inferior point”. Lastly, the “superior point” was comprised of the B-scan that measured 400 µm above the “inferior point”. To determine the overall total retinal and sublayer thickness for each study eye, measurements were made at each of these reference points to both the left and right of the ONH (Fig 1). These six measurements were then averaged together to acquire final retinal thickness values.

### Histological Evaluation

**Figure 2:**
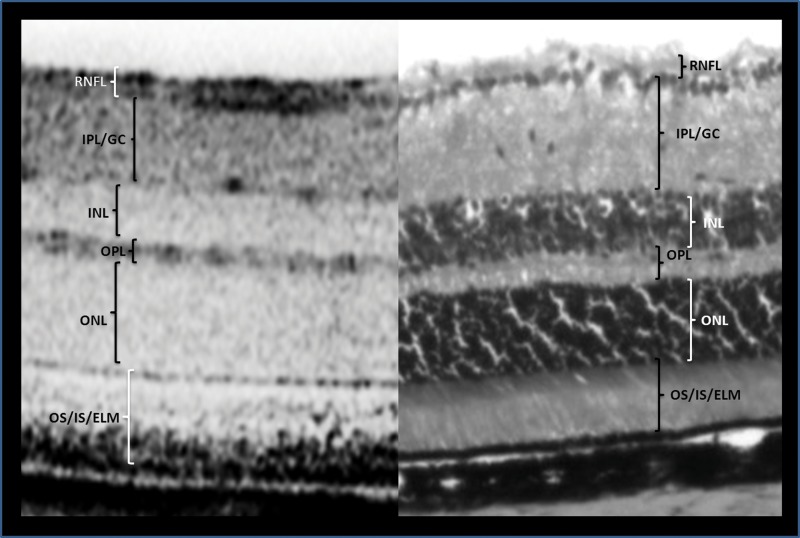
Cross-sectional views of the C57BL/6 mouse retina by SD-OCT (left) and histology (right). OS/IS/ELM, outer segment/inner segment/external limiting membrane; ONL, outer nuclear layer; OPL, outer plexiform layer; INL, inner nuclear layer; IPL/GC, inner plexiform layer/ganglion cell; RNFL, retinal nerve fiber layer.

Animals were euthanized with CO_2_ inhalation followed by cervical dislocation. The same eyes that had SD-OCT scans were enucleated and then punctured with a 30-gauge needle and submerged into 4% paraformaldehyde (PFA) for 2–4 hours. This was followed by a series of submersions into phosphate buffered saline (PBS) with increasing concentrations of sucrose in the following manner: PBS with 5% sucrose (6 hrs), PBS with 10% sucrose (10 hrs), and PBS with 20% sucrose (10 hrs). Samples were then removed from the PBS with sucrose solution and solidified into optimal cutting temperature compound in a container with dry ice and 2-methylbutane. A cryostat microtome was used to produce sample slices of 10 µm per section. Every twentieth section was selected for tissue staining. The samples were then stained with H&E using standard laboratory protocols. The Zeiss Axioskop 2 Mot Plus (Carl Zeiss MicroImaging, Inc., Thornwood, NY) microscope, with 10x objective lens, was used to evaluate histological sections. All sections selected for light microscopy evaluation contained optic nerve tissue landmarks. The histological section that first demonstrated the appearance of the ONH was considered the histological ‘inferior point’. As illustrated in Figures 1 and 2, histological measurements of retinal sublayers were similarly performed as mentioned for SD-OCT scan thickness measurements. The manual caliper instrument, from the Axiovision 4.8 (Carl Zeiss MicroImaging, Inc., Thornwood, NY) imaging software, was used to measure histological sublayer retinal thickness. TRT was derived from the sum of all measured histological layers up to but not including the retinal pigment epithelium.

### Statistical Analysis

Mean retinal thickness values, for each study eye, were acquired by averaging the thickness values obtained from the retinal layers left and right of the ONH corresponding to the zero point, 200 µm, and 400 µm locations. Overall mean and standard error of the mean (± SE) for the retinal layers were calculated from the sample observations for thickness values for each retinal layer obtained from the eight animals evaluated (n = 48). Correlation analysis (GraphPad Software Inc., La Jolla, CA) was used to assess the association between thickness measurements from histology and SD-OCT images. The statistical significance of correlations for the TRT and retinal sublayers was achieved if the p-values were less than 0.05.

## Results

The mean thickness of each retinal sublayer and TRT in each animal measured by SD-OCT and histology is depicted in Table 1. The overall mean retinal thickness of individual sublayers and TRT when measured by the two methods is displayed in Table 2. The overall TRT by SD-OCT evaluation was 210.95±1.09 µm while TRT measurement from histology was 219.58±2.67 µm.

**Table 1:** Mean SD-OCT and histology retinal thickness (in µm) for each study animal.

Animals	OS/IS/ELM	ONL	OPL	INL	IPL/GC	RNFL	TRT
**SD-OCT**							
Animal 1	42.33	59.08	15.92	26.83	51.28	15.20	210.65
Animal 2	41.38	60.05	13.95	25.48	52.58	13.17	206.62
Animal 3	43.72	57.80	14.87	24.23	53.60	15.82	210.03
Animal 4	41.77	64.50	15.88	23.62	55.28	15.52	216.57
Animal 5	38.55	63.78	16.63	23.47	51.70	16.65	210.78
Animal 6	39.87	58.05	16.87	25.13	52.27	16.17	208.35
Animal 7	40.23	61.65	16.82	23.95	52.43	15.77	210.85
Animal 8	44.55	61.10	15.45	25.52	51.40	15.78	213.80
**Histology**							
Animal 1	48.33	53.33	20.00	31.67	66.67	15.00	225.00
Animal 2	41.67	53.33	18.33	35.00	63.33	13.33	210.00
Animal 3	48.33	55.00	20.00	36.67	71.67	16.67	216.67
Animal 4	38.33	51.67	15.00	33.33	65.00	11.67	211.67
Animal 5	45.00	58.33	16.67	38.33	68.33	11.67	223.33
Animal 6	60.00	51.67	18.33	35.00	58.33	11.67	228.33
Animal 7	38.33	51.67	18.33	36.67	70.00	16.67	213.33
Animal 8	55.00	51.67	20.00	38.33	61.67	11.67	228.33

(n = 48).

SD-OCT, spectral domain optical coherence tomography; OS/IS/ELM, outer segment/inner segment/external limiting membrane; ONL, outer nuclear layer; OPL, outer plexiform layer; INL, inner nuclear layer; IPL/GC, inner plexiform layer/ganglion cell; RNFL, retinal nerve fiber layer; TRT, total retinal thickness.

**Table 2:** Mean (± SE) SD-OCT/histology retinal thickness and calculated ‘correlation constant’ for each retinal sublayer.

Layer	SD-OCT (µm)	Histology (µm)	SD-OCT/Histology
Outer Segment/Inner Segment/External Limiting Membrane/	41.55 (±0.60)	46.88 (±1.43)	0.91
Outer Nuclear Layer	60.75 (±0.70)	53.33 (±0.75)	1.14
Outer Plexiform Layer	15.80 (±0.36)	18.33 (±0.54)	0.87
Inner Nuclear Layer	24.78 (±0.60)	35.63 (±0.72)	0.70
Inner Plexiform Layer/Ganglion Cell	52.57 (±0.67)	65.63 (±1.26)	0.80
Retinal Nerve Fiber Layer	15.51 (±0.43)	13.54 (±.70)	1.17
Total Retinal Thickness	210.95 (±1.09)	219.58 (±2.67)	0.96

SD-OCT, spectral domain optical coherence tomography.

Table 2 also exhibits the correlation constants for retinal thickness (TRT and individual sublayers) as the ratio of measurements from SD-OCT scan sections to histological sections. The mean ‘correlation constant’ for TRT, when comparing mean SD-OCT TRT to mean histology is 0.96. The variance (± SD  = 0.04) in the mean TRT measurement ratio was small (Table 3).

**Table 3:** Correlation constant (SD-OCT/histology) for total and retinal sublayer thickness measurements in each animal.

Animals	OS/IS/ELM	ONL	OPL	INL	IPL/GC	RNFL	TRT
Animal 1	0.88	1.11	0.80	0.85	0.77	1.01	0.90
Animal 2	0.99	1.13	0.76	0.73	0.83	0.99	0.92
Animal 3	0.90	1.05	0.74	0.66	0.75	0.95	0.85
Animal 4	1.09	1.25	1.06	0.71	0.85	1.33	1.01
Animal 5	0.86	1.09	1.00	0.61	0.76	1.43	0.88
Animal 6	0.66	1.12	0.92	0.72	0.90	1.39	0.89
Animal 7	1.05	1.19	0.92	0.65	0.75	0.95	0.91
Animal 8	0.81	1.18	0.77	0.67	0.83	1.35	0.90
Average	0.91	1.14	0.87	0.70	0.80	1.17	0.96
Standard Dev.	0.14	0.06	0.12	0.07	0.06	0.22	0.04

SD-OCT, spectral domain optical coherence tomography; OS/IS/ELM, outer segment/inner segment/external limiting membrane; ONL, outer nuclear layer; OPL, outer plexiform layer; INL, inner nuclear layer; IPL/GC, inner plexiform layer/ganglion cell; RNFL, retinal nerve fiber layer; TRT, total retinal thickness.

**Figure 3:**
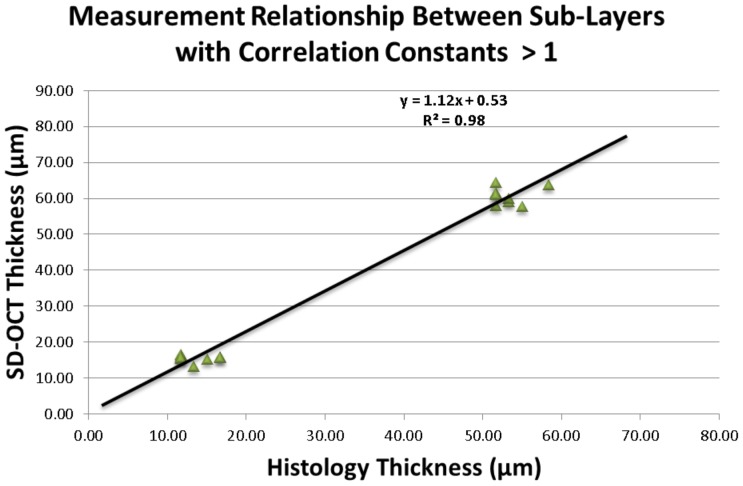
Association between mean SD-OCT and histological thickness with correlation constants >1. Scatter plot points refer to all observations for outer nuclear layer and retinal nerve fiber layer.

**Figure 4:**
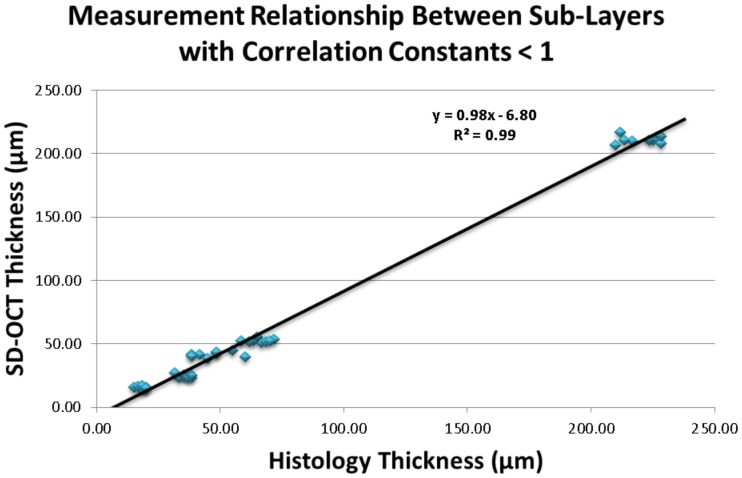
Association between mean SD-OCT and histological thickness with correlation constants <1. Scatter plot points refer to all observations for outer segment/inner segment/external limiting membrane, outer plexiform layer, inner nuclear layer, inner plexiform/ganglion cell layers, and total retinal thickness.

Sublayer analyses showed that the ‘correlation constant’ of SD-OCT to histology thickness was greater than one for the ONL (1.14) and RNFL (1.17) and was less than one for the OS/IS/ELM (0.91), OPL (0.87), INL (0.7), and IPL/GC (0.8) layers (Tables 2&3). In general, thickness measurement comparisons between SD-OCT and histology demonstrated significant correlations. The ONL and RNFL, with ‘correlation constants’ greater than one, showed a highly significant strong correlation (r^2^ = 0.98, p<0.01) [Figure 3]. Similarly, for the layers with ‘correlation constants’ less than one, the relationship exhibited between SD-OCT and histological measurements was also significantly correlated (r^2^ = 0.99, p<0.01) [Figure 4].

## Discussion

In retinal diseases, ocular histology is still considered to be the gold standard for assessing structural morphology. Histology provides a snapshot view of the microstructural environment of the retina by staining and tissue preservation techniques. The drawback of histology in humans is that it is difficult to conduct longitudinal studies to describe the course of a disease process. However, in animal studies, the animals can be sacrificed sequentially to get longitudinal data to improve our understanding of many retinal disorders.

Technological innovation in ophthalmic imaging has led to *in vivo* OCT, possibly replacing histology for assessing retinal morphology. Over time, OCT technology has advanced from the time-domain (TD-OCT) capability of the Stratus (Carl Zeiss Meditec) to the SD-OCT features of the Cirrus (Carl Zeiss Meditec) and Spectralis (Heidelberg Engineering, Inc.), with better speed and resolution. The advent of higher bandwidth SD-OCT imaging devices has allowed for single digit micrometer resolution potentials, which show comparable anatomical delineation as histology [7].

With the possible shift from histological biopsies to optical biopsies with OCT, several studies have reported normative data for macular retinal thickness using TD-OCT [8] and the SD-OCT [9] devices. Furthermore, there have been some reports comparing OCT retinal thickness measurements to histological measurements. In one study the authors used TD-OCT to ‘qualitatively’ correlate macular retinal thickness scans to histology in three patients with exenterated cancerous orbits [10]. They reported some level of association between high magnification light microscopic histologic images to lower resolution TD-OCT scans, but no ‘quantitative’ measurements were made [10]. Similarly, another study utilized *ex vivo* SD-OCT macular scans of donor eyes, prior to histological processing, to determine the extent of volumetric changes associated with histology [11]. They reported a median tissue shrinkage of 14.5% overall with 29% in the foveal area. In yet another maturation study of the human retina, SD-OCT morphology and retinal layer thickness measurements from 22 premature infants, 30 term infants, 16 children and one adult were qualitatively contrasted with light microscopy histologic images from age-matched donors [12]. Their findings suggested that at all ages, SD-OCT images demonstrated high agreement with histology.

In animal studies too, although histological measurements are still the gold standard for retinal morphological evaluation, there has been a transition of retinal disease animal model research towards *in vivo* imaging [13]–[15]. Moreover, studies utilizing SD-OCT have provided useful information on longitudinal changes resulting from retinal disease pathophysiology [14], [16]. The reproducibility, reliability, and non-invasive qualities of this technology make it ideal for use in clinical as well as research applications [17]–[19]. Since different histological fixation techniques can be used and also since different OCT instruments can be utilized in different studies, it is not advisable to compare absolute values of TRT or its sublayers. Instead, a ratio in the form of a ‘conversion constant’ would be more useful and accurate. There are no studies that establish a ‘conversion constant’ to show the relationship between OCT and histological retinal thickness measurements.

The present study demonstrated retinal thickness measurement correlations between SD-OCT assisted optical biopsy to histological methods in a C57BL/6 mouse model. Histological measurements for the total retinal thickness and its sublayers were compared to the SD-OCT measurements in the same eyes at similar locations to generate a ‘conversion constant’. We determined that the mean and standard deviation conversion constant was approximately 0.96±0.04 for the TRT. We also determined the different retinal sub-layer ‘conversion constants’, where the ratio of retinal thickness by SD-OCT to histology ranged from 0.70 to 1.17 (Table 2). It seemed that the variation in this constant was less in outer retinal layers (0.87–1.14) as compared to inner retinal layers (0.70–1.17).

There are very few studies [14], [15], [20] that have compared the retinal thickness, as measured by histology vs. OCT, in C57BL/6 mice models (Table 4). Even those studies did not use an elaborate point-to-point methodology as the present study. Two of the three studies [14], [20] were from the same lab. The third study [15], although performed on C57BL/6 mice, compared the retinal thickness by OCT and histology after artificially creating a retinal detachment.

**Table 4:** Comparison of SD-OCT/histology ‘correlation constant’ between present and prior studies.

Layer	Ferguson et al.	Fischer et al.	Huber et al.	Cebulla et al.
Outer Segment/Inner Segment/External Limiting Membrane/	0.91	0.94	…	0.50
Outer Nuclear Layer	1.14	1.10	…	0.58
Outer Plexiform Layer	0.87	…	…	…
Inner Nuclear Layer	0.70	0.77	…	…
Inner Plexiform Layer/Ganglion Cell	0.80	0.88	…	…
Retinal Nerve Fiber Layer	1.17	…	…	…
Total Retinal Thickness	0.96	0.93*	1.05*	0.66*

*The correlation constants in all prior studies were not reported but calculated by ‘extrapolating’ from the data presented in those studies.

The study by Fischer et al. [20] compared retinal thickness measurements by OCT and histology in the similar mice model as well as other retinal degeneration models. They utilized a third generation SD-OCT instrument (Heidelberg Engineering, Germany) for the measurements. It seems that nine data points were used (extrapolating from their Figure Two A [20]) but it is not clear whether these were nine points from different sites of the same animal or 9 different animals were used. They also do not mention whether they averaged measurements at each point or only one measurement was performed. Unlike the present study, the comparison of histology and OCT were not performed at similar sites. Nevertheless, they found a significant correlation coefficient of 0.89 for the total retinal thickness between the two methodologies. They also reported a close agreement between histology and OCT for the retinal sub-layers they measured (ILM-IPL, INL, OPL, ONL and IS/OS layers). Although quantitative measurements are not reported, extrapolating from their figure (Figure Two B [20]), it seems that the TRT was in the range of ≈245 µm by histology and ≈230 µm by SD-OCT, leading to a ‘conversion constant’ of ≈0.94. This is similar to the ‘conversion constant’ of 0.96 in the present study. The sub-layer analyses in the study were performed on five sublayers and were very similar to that in our study (Table 4). The tissue fixative used in this histological study was 2.5% gluteraldehyde as compared to PFA in our study.

The study by Huber et al. [14] utilized the HRA + SD-OCT (Heidelberg, Germany) device and investigated its efficacy to study mouse models of retinal degeneration as compared to the C57BL/6 mice (n = 37). Although the SD-OCT was done on 37 mice, only three of these animals were sacrificed to study their histological structure. The measurement of TRT was based on similar points of the retina. They reported a TRT of 237±2 µm by SD-OCT whereas they do not report the TRT by histology. However, extrapolating from their figure (Figure Six C [14]), it seems that the TRT was in the range of 225 µm by histology. That would calculate the approximate ‘conversion constant’ to be 1.05 (Table 4). They do report that they obtained a high correlation of TRT between SD-OCT and histology measurements (R^2^ = 0.897). It is interesting to note that whereas the TRT in the present study was in the range of 210.95 µm by SD-OCT, it was reported to be 237 µm by Huber et al. This difference could possibly be due to the instrumentation – the Heidelberg SD-OCT measures the TRT, inclusive of the retinal pigment epithelium layer (RPE) whereas the Bioptigen SD-OCT (used in the present study) does not include the RPE sub-layer. In the sub-layer analyses, they also reported that the correlation coefficient was even higher for the outer retinal thickness (R^2^ = 0.978). This was similar to what was observed in the present study. The fixative used in this study was also 2.5% gluteraldehyde as compared to PFA in our study.

The study by Cebulla et al. [15] reported longitudinal data on the SD-OCT measurements of the retinal thickness in a C57BL/6 model where retinal detachment was artificially created. In the same model, they also compared the TRT by SD-OCT and histology. They utilized a custom-made ultra high resolution imaging system and created retinal detachment in 17 mice. Although they do not report the TRT by SD-OCT or histology, extrapolation from their figures (Figures Five A and Five B [15]) show that the TRT by SD-OCT and histology were approximately 210 µm and 320 µm, respectively. This would calculate the conversion constant as approximately 0.66, much lower than found in the present study or the other studies [14], [20]. However, they stated that the imaging system used measured the retinal thickness from nerve fiber layer to the base of the photoreceptor outer segment. Moreover, they used a retinal detachment model despite calculating the TRT in the attached part of the retina. They used the same fixative as our study (PFA).

The present study and some of the other studies in the past have shown a good correlation between the measurements obtained by optical section (SD-OCT) and histological sections. The observed small differences in values between the *ex vivo* and *in vivo* measurements could be due to multiple reasons. Histological artifacts such as shrinkage, dehydration, and swelling can contribute to changes in the retinal structure as histological sections are prepared. This can be due to the choice of fixative which can also lead to artifacts. Also, when measuring retinal thickness by SD-OCT or histology with algorithms dependent on demarcating retinal layers, the precision of the measurement tool affects the reliability of the measurement [21]. In the present study, the caliper used for light microscopic measurement was calibrated to a resolution limit of 10 µm. This could have hampered our ability to accurately measure thinner layers such as the RNFL. The resolution capability of the SD-OCT unit also plays a role when defining and measuring retinal thickness. The Bioptigen Envisu R 2200 SDOIS commercial unit used for this study has a tissue axial resolution of approximately 1.7 mm. This resolution power was not enough to accurately define deeper layers such as the RPE. Although RPE measurements were available in histological sections, corresponding measurements were not available for SD-OCT images. For this reason, as this was a comparative study, we did not include RPE measurements in our study, both in the histology as well as the SD-OCT measurements.

The present study has established a ‘conversion constant’ of 0.96 for the measurement of retinal layer thickness between high-resolution SD-OCT optical biopsies to that of light microscopy histology. This study suggests that for all practical purposes, the retinal thickness, as measured by SD-OCT is equivalent to gold standard histological biopsy measurements. SD-OCT is more convenient, inexpensive, and requires no animals to be sacrificed, as for histology. In the era of growing utilization of SD-OCT technology and continued improvement of its resolution, this study can influence the design of future vision science research with animal models when analyzing longitudinal disease pathology as well as in monitoring the effects of pharmacological interventions on retinal disease processes.
